# Efficacy of liraglutide in patients with diabetic nephropathy: a meta-analysis of randomized controlled trials

**DOI:** 10.1186/s12902-022-01006-6

**Published:** 2022-04-07

**Authors:** Niroj Mali, Feng Su, Jie Ge, Wen Xing Fan, Jing Zhang, Jingyuan Ma

**Affiliations:** 1 Department of Nephrology, the First Affiliated Hospital of Kunming Medical University, Kunming, 650032 Yunnan Province China; 2 NHC Key Laboratory of Drug Addiction Medicine, the First Affiliated Hospital of Kunming Medical University, Kunming, 650032 Yunnan Province China; 3 Department of Nutrition, the First Affiliated Hospital of Kunming Medical University, No.295, Xichang Road, Kunming, 650032 Yunnan Province China

**Keywords:** Type 2 diabetes mellitus (DM), Liraglutide (Lira), Diabetic nephropathy, Meta-analysis

## Abstract

**Background:**

The efficacy of liraglutide to treat type 2 diabetic nephropathy (T2DN) remains controversial. Thus, we conducted this meta-analysis to systematically evaluate the clinical effect of liraglutide on T2DN patients.

**Methods:**

Eight databases (PubMed, Web of Science, the Cochrane Library, EMBASE, Chinese National Knowledge Infrastructure (CNKI), Wanfang database, China Science and Technology Journal Database, and China Biology Medicine Database (CBM)) were searched for published articles to evaluate the clinical efficacy of liraglutide in subjects with T2DN. The Revman 5.3 and Stata 13 software were used for analyses and plotting.

**Results:**

A total of 18 randomized controlled trials (RCTs) with 1580 diabetic nephropathy patients were screened. We found that the levels of UACR, Scr, Cysc were lower in the experimental group of T2DN patients treated with liraglutide than in the control group intervened without liraglutide. Liraglutide also reduced the levels of blood glucose (including FBG, PBG, and HbA1c), body mass index (BMI), and anti-inflammatory indicators (TNF–α, IL-6). However, there was no significant difference in BUN and eGFR between the experimental group and the control group.

**Conclusions:**

Liraglutide reduced the levels of Blood Glucose, BMI, renal outcome indicators, and serum inflammatory factors of patients with T2DN, suggesting the beneficial effects of liraglutide on renal function.

## Background

Although primary prevention for risk factors and early intervention in the levels of blood glucose effectively reduce the rates of incidence and renal failure caused by type 2 diabetic nephropathy (T2DN), T2DN is still one of the most serious and prevalent microvascular complications of diabetes mellitus with type 2 (T2DM) worldwide [1]. The number of adult diabetes patients in the world obtained from the report of the international diabetes federation is about 536.6 million (approximately 10.5% of the world population) by the end of 2021, which is expected to reach 783.2 million (12.2%) in 2045 [2]. Unfortunately, more than 40% of individuals with diabetes mellitus will develop kidney disease over time [3]. The morbidity of T2DM is exceedingly high, and an increasing number of T2DN cases are relatively detected. In fact, T2DN has become the major cause leading to end-stage renal disease (ESRD) [4].

For a long time in the past, a wide variety of drugs such as insulin, metformin, sulfonylurea, meglitinide, and thiazolidinedione were utilized to control the level of blood glucose and reduce the risk of diabetic complications [5]. However, substantially adverse effects associated with these traditional drugs including hypoglycemia and weight gain, and drugs-resistance lead to the limitation of drug application. Fortunately, a new kind of compound glucagon-like peptide 1 receptor agonists (GLP-1RA) have been widely used in treatments of diabetes and its associated complications in recent years. Liraglutide, a common GLP1-RA, shows a good curative effect in reducing weight and controlling blood glucose. Notably, liraglutide is likely to have a considerable renoprotective effect [6, 7].

Some previous studies have shown that liraglutide can reduce urine protein and has a renal protective effect [8, 9]. But some others did not find the same results [10]. In conclusion, the efficacy of liraglutide to treat T2DN remains controversial. Thus, this meta-analysis aims to estimate the efficacy of liraglutide in the treatment of T2DN and which related indicators may be affected.

## Materials and methods

### Search strategy

Health-related electronic databases including PubMed, Embase, Web of Science, the Cochrane Library, CNKI, Wanfang Database, VIP, and CBM Database, were searched to identify eligible studies to July 2021 inclusive, using the following Medical Subject Heading (MeSH) AND/OR entry words in any field, “diabetic nephropathy”, “diabetic nephropathies”, “proteinuria”, “diabetic kidney disease”, “albuminuria”, “liraglutide”, “diabetic glomerulosclerosis”, “urinary albumin excretion”, “type 2 diabetes mellitus”. Correspondingly, the search strategy was slightly adjusted according to the range of the search results in different databases. In addition, the related research was limited to RCT published in English or Chinese language. To avoid missing other relevant articles, we also manually retrieved the references of every article and relevant reviews to investigate any additional eligible studies.

### Inclusion criteria

Study inclusion criteria were as follows:

1. RCTs (randomized controlled trials) were used in these research;
2. Studies including patients with type 2 diabetes with nephropathy;
3. Patients who were on a strict diet and exercise regimen were included in the studies, some were prescribed with anti-hypertensive medications or other anti-hyperglycemic medications (control group), while others were prescribed with liraglutide (experimental group);
4. Studies that reported renal function outcomes, including estimated glomerular filtration rate (eGFR); urine albumin creatinine ratio (UACR); Blood Urea Nitrogen (BUN); serum creatinine (Scr); serum cystatin C (CysC);

### Data extraction

Based on the inclusion and exclusion criteria, all screened studies were independently identified by two reviewers (NM and FS). Notably, any discrepancy between the two reviewers was resolved by discussion or the third reviewer (FWX). Then, the selected full-text articles were performed eligibility evaluation to determine whether they were suitable for the current meta-analysis. Furthermore, the information extracted from each study included:

1. the basic information, including the initial author’s name, the year of publication, the type of study, and the total number of patients, gender composition, the average age of patients in the experimental group and the control group, the therapeutic approaches, course of treatment.
2. the evaluation index of the outcome included: hypoglycemic related indicators (FBG, PBG, HbA1c) and BMI; renal function(UACR, Scr, CysC, BUN, eGFR); anti-inflammatory indicators (IL-6, TNF-α).

### Assessment of quality of evidence

The quality evaluation for every selected article was independently assessed by two authors (MN and SF) using the Cochrane Collaboration’s tool for assessing the risk of bias [11]. In general, the evaluation contents included the quality appraisal of the literature comprised random sequence generation (Selection Bias), allocation concealment (Selection Bias), blinding of participants and personnel (Performance Bias), blinding of outcome assessment (Detection Bias), incomplete outcome data (Attrition Bias), and selective reporting (Reporting Bias), and other sources of bias. Similarly, disagreements for the risk of bias by two investigators were resolved by discussing or the third reviewer. Markedly, articles that had clearly defined details and met or surpassed the quality criteria were defined as low-risk; if not, they were deemed high-risk. Ambiguous articles concerning quality criteria remained deemed to be of unclear risk. Notably, the quality of trials was evaluated using the Cochrane Collaboration’s tool for evaluating the risk of bias in randomized controlled trials; quality was not used as a standard for the selection of trials, however merely for descriptive purposes.

### Data analysis

Review Manager Software 5.3.5 (RevMan 5.3.5) and Stata 13 Software were used for all data analyses and plotting. The Chi-Squared-based Q-tests and I-squared (I2) statistics were utilized to evaluate the statistical heterogeneity of the included studies [12]. The value of I2 test greater than 50% and *p* ≤ 0.05 were regarded as substantial heterogeneity. Then DerSimonian-Laird random-effect model [13] was performed, otherwise, a Mantel-Haenszel fixed-effect model [14] was conducted. Subsequently, the statistical significance of Standardized Mean Difference (SMD) or Weighted Mean Difference (WMD), and 95% confidence intervals (95% CIs) were estimated by Z tests. In addition, the symmetry of funnel plots was applied to determine the publication bias of the selected studies [15]. Sub-group analyses were carried out by HbA1c and ACR due to significant heterogeneity across the included studies.

## Results

### Literature search and selection

**Fig. 1:**
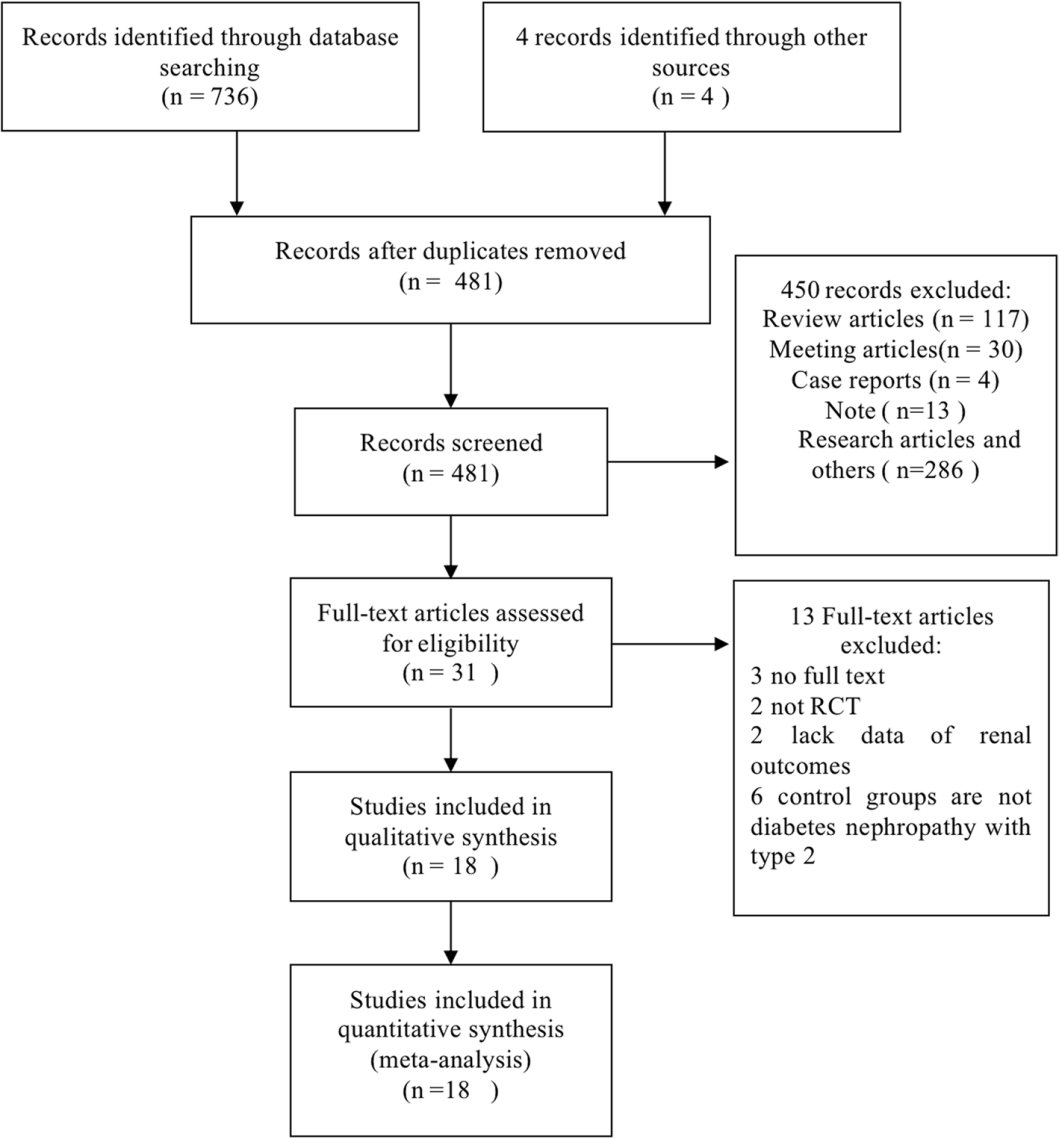
The PRISMA flow diagram of study selection

A total of 740 articles including 736 records from the electronic databases using different search strategies and 4 articles through literature tacking and reading were identified. After removing 481 duplications and 450 records unmet the inclusion criterion, 31 studies were selected to further verify through full-text reading. In the residual records, 13 full-text articles were excluded with reasons (*n* = 13) as follows: 2 trials are not RCT, 3 articles had no full text that could be found to extract data, 2 lack renal outcomes data, 6 control groups are not DN with type 2. Finally, 18 articles that satisfied the inclusion criteria were included in the meta-analysis. The procedure of finding articles and choosing studies was demonstrated in Fig. 1.

### Characteristics of eligible studies

In our meta-analysis, all of the selected studies were published from 2014 to 2020. In detail, 18 studies [16–33] included 1580 DN patients enrolled in the study, of which 786 were in the liraglutide group and 794 were in the control group. Among these studies, 12 articles [17–21, 23, 24, 26–28, 30, 32, 33] illustrated the average age of patients with T2DN and the ratio of sex, while only 5 RCTs [17, 20, 23, 26, 32] showed the course of disease in patients with T2DM. Besides, in the liraglutide group, liraglutide was given a duration of 4 to 24 weeks with a dosage of 0.6 to 1.8 mg/day. However, kinds of drugs from different studies included trials placebo, routine treatment, Huangkui capsules, nephritis rehabilitation tablets, insulin or active comparators (metformin, glimepiride, and glargine) were used in the control group. The detailed characteristics of the included studies were listed in Table 1.

**Table 1:** Characteristics of the studies involved

Research	Sample	Age/years		Sex ratio (males/females)		Course of T2DM/years		Interventions		Duration	Outcomes
	T/C	T	C	T	C	T	C	T	C		
Zha 2018 [16]	30/30	–	–	–	–	–	–	LIR(0.6 to 1.8 mg qd ih) plus Huang kui capsule	Huangkui Capsules 2.5 g tid po	2 months	ACDF
Cao 2020 [17]	30/30	55.21 ± 6.32	56.12 ± 6.92	17/13	15/15	4.08 ± 1.74	4.19 ± 1.52	LIR(0.6 to 1.2 mg qd po) plus nephritis rehabilitation tablets	nephritis rehabilitation tablets 0.48 g/tablet, 5tablets/time, tid	12 weeks	ABCGHIJK
Dong 2018 [18]	43/43	53.7 ± 6.2	54.6 ± 8.7	20/23	23/20	–	–	LIR(0.6 to 1.8 mg qd ih) + INS	INS	6 months	ABCDEFGH
Chen 2016 [19]	30/31	–	–	–	–	–	–	LIR(0.6 to 1.8 mg qd ih) + RT	RT	24 weeks	ACDEH
Hu Yanyun 2018 [20]	55/55	59.64 ± 6.51	59.66 ± 6.54	35/20	37/18	6.28 ± 1.23	6.39 ± 1.14	LIR(0.6 to 1.2 mg qd ih)plus RT	RT	8 weeks	ABCHK
Ren Lijuan 2019 [21]	15/15	44.8 ± 2.7	51.3 ± 2.9	7/8	9/6	–	–	LIR (0.6 to 1.8 mg tid po) plus huangkui capsules	Huangkui Capsules 2.5 g tid	6 months	ABCF
Ren Wei 2015 [22]	24/24	–	–	–	–	–	–	LIR(0.6 to 1.8 mg qd ih) + RT	RT	6 months	ACDFH
Shi 2019 [23]	30/30	57.32 ± 3.69	57.63 ± 3.12	17/13	18/12	7.13 ± 2.24	7.08 ± 1.71	LIR (0.6 to 1.2 mg qd ih) plus Benazepril 10 mg qd	Benazepril 10 mg qd	10 weeks	CHJKH
Yang 2016 [24]	100/100	66.8 ± 14.7	67.4 ± 13.5	45/55	48/52	–	–	LIR (0.6 to 1.2 mg qd ih) plus Telmisartan 40 mg qd	Telmisartan 40 mg qd	10 weeks	CHJK
Zhao 2014 [25]	19/26	–	–	–	–	–	–	LIR (0.6 to 1.8 mg qd ih) plus Valsartan	Valsartan 80 mg qd	6 months	CDI
Zheng 2015 [26]	110/110	58 ± 4.9	57 ± 5.1	67/43	65/45	6.9 ± 2.8	7.1 ± 2.4	LIR (0.6 to 1.2 mg qd ih) plus INS	INS	4 weeks	ADGH
Hu Linlin 2018 [27]	30/30	42.5 ± 11.6	41.3 ± 10.7	18/12	17/13	–	–	LIR(0.6 to 1.8 mg tid po) plus huangkui capsules	Huangkui Capsules 2.5 g tid po	6 months	ACDF
Aiyitan 2017 [28]	89/73	58.1 ± 8.1	57.8 ± 7.9	49/40	39/34	–	–	LIR(0.6 to 1.8 mg qd ih) plus RT	RT	8 weeks	ABF
Shen 2017 [29]	30/30	–	–	17/13	16/14	–	–	LIR (0.6 to 1.2 mg qd ih) pluse Olmesartan 20 mg/d	Olmesartan 20 mg/d	6 months	ACFHJK
Liu Rui 2016 [30]	59/75	57.5 ± 7.4	58.2 ± 7.9	33/26	43/32	–	–	LIR (0.6 to 1.2 mg qd ih) plus RT	RT	8 weeks	ABF
Liu Chuyv 2015 [31]	13/13	–	–	–	–	–	–	LIR (0.6 to 1.2 mg qd ih) + Olmesartan	Olmesartan 20 mg qd + INS	6 months	DFI
Li 2017 [32]	21/21	48.2 ± 9.0	49.1 ± 8.3	11/10	13/8	4.2 ± 1.1	7.38 ± 1.5	LIR plus RT	RT + INS	12 weeks	ABCDEGH
Jian 2018 [33]	58/58	56.51 ± 6.05	56.33 ± 8.63	32/26	29/29	–	–	LIR (0.6 mg to 1.2 mg qd ih) plus Metformin	Metformin 1 g bid	3 months	ABCDFHJK

*LIR* liraglutide, *RT* routine treatment, *INS* insulin

A. FBG

B. PBG

C. HbA1c

D. BMI

E. eGFR

F. UACR

G. BUN

H. Scr

I. Cysc

J. IL-6

K. TNF-α

### Risk of bias

**Fig. 2:**
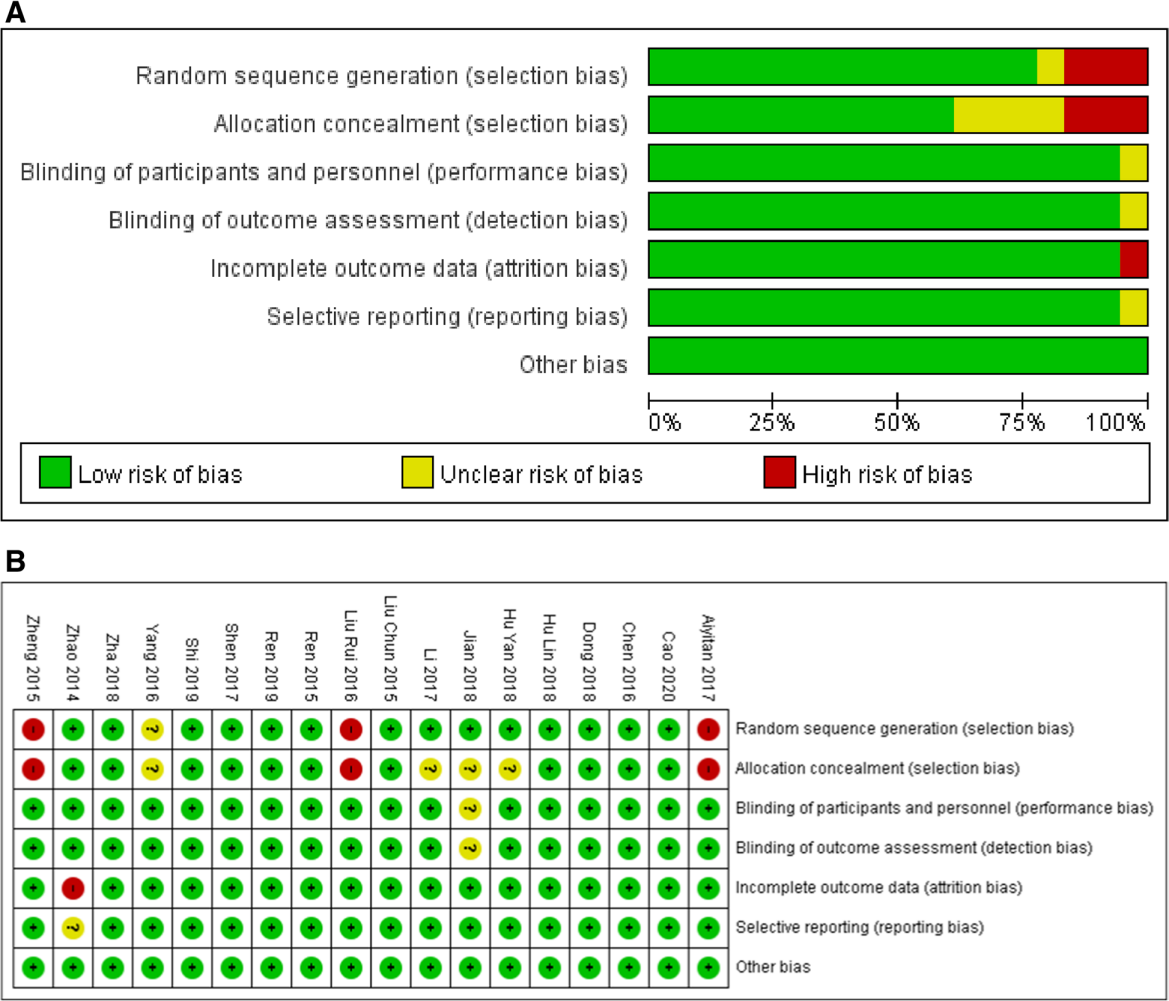
Risk of bias graphs and summaries in several categories through all of the studies involved. **A** Risk of bias graph; **B** risk of bias summary

The Cochrane Collaboration’s tool was used to evaluate the quality of the individual studies based on the randomization, allocation hiding, blinding, publication bias, etc. Among 18 studies, 14 of them were described as randomized trials (5 of which were random number table method), 1 study was grouped according to the patient’s wishes, 2 studies were grouped according to the patient’s original treatment, and the last one article has not mentioned the method of grouping. For allocation concealment, 4 studies were not mentioned, and 3 studies may have higher allocation hidden risk. In addition, one trial was a single-blind experiment, and the others were not indicated the method of blinding. Unfavorably, one article had incomplete primary outcome indicators and selective-publication possibility. Notably, no other bias factor was found in all articles. The specific quality evaluation chart was shown in Fig. 2A-B.

## Effect of interventions

### Relationship of liraglutide with renal function

**Fig. 3:**
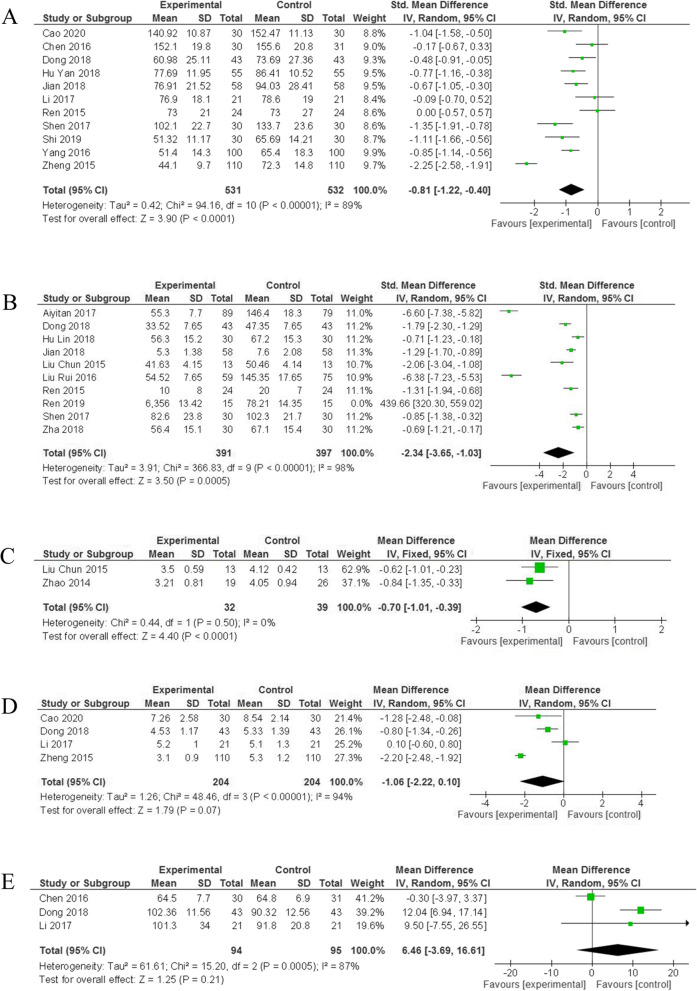
Forest plots for the effects on renal function of liraglutide in patients diabetic nephropathy. **A** Scr; **B** UACR; **C** CysC; **D** BUN; **E** eGFR. SMD, standard mean difference; WMD, weight mean difference; CI, confidence interval; IV, inverse variance; df, degrees of freedom; green squares, an effect size of each study; the size of green squares, the weight of each study; Black diamonds, test for overall effect; horizontal lines, confidence intervals

To estimate the effect of liraglutide on renal function in patients with T2DN, the statistic differences of eGFR, BUN, Scr, UACR, and CysC between the liraglutide group and the control group were calculated and visualized by forest maps. The results of our meta-analysis suggested that there were significant differences between the liraglutide group and the control group in the levels of Scr (SMD = -0.81, 95% CI: [− 1.22,-0.4], *p* < 0.0001) (Fig. 3A, Table 2), UACR (SMD = -2.34, 95% CI: [− 3.65, − 1.03], *p* = 0.0005) (Fig. 3B, Table 2) and CysC (WMD or MD = -0.70, 95% CI: [− 1.01, − 0.39], *p* < 0.0001) (Fig. 3C, Table 2) after treatment. However, no differences between the liraglutide group and the control group were detected in the levels of BUN (WMD = -1.06, 95% CI: [− 2.22,0.10], *p* = 0.07) (Fig. 3D, Table 2) and eGFR(WMD = -0.81, 95% CI: [− 1.22, − 0.40], *p* = 0.21) (Fig. 3E, Table 2). In summary, liraglutide greatly reduced the levels of UACR, Scr, and Cysc compared with treatment without liraglutide.

**Table 2:** Study findings summary

Outcome	Number of studies	Sample	Heterogeneity			Analysis model	Statistical method	WMD/SMD (95% CI)	*P*-value
		Test/Control	χ^2^	I^2^	*P*				
FBG	14	624/625	141.13	91%	< 0.00001	Random-effects	Inverse Variance	−0.66[− 1.04,-0.27]	0.0009
PBG	8	370/370	12.94	46%	0.07	Fixed-effects	Inverse Variance	−1.51[− 1.68,-1.34]	< 0.00001
HbA1c	14	515/523	217.29	94%	< 0.00001	Random-effects	Inverse Variance	−0.61[− 0.95,-0.27]	0.0004
BMI	10	378/386	98.99	91%	< 0.00001	Random-effects	Inverse Variance	−2.27[−2.98,-1.56]	< 0.00001
eGFR	3	94/95	15.20	87%	0.0005	Random-effects	Inverse Variance	6.46[−3.69,16.61]	0.21
UACR	10	391/397	366.83	98%	< 0.00001	Random-effects	Inverse Variance	−2.34[−3.65,-1.03]	0.0005
BUN	4	204/204	48.46	94%	< 0.00001	Random-effects	Inverse Variance	−1.06[−2.22,0.10]	0.07
Scr	11	531/532	94.16	89%	< 0.00001	Random-effects	Inverse Variance	−0.81[−1.22,-0.4]	< 0.0001
CysC	2	32/39	0.44	0%	0.5	Fixed-effects	Inverse Variance	−0.7[−1.01,-0.39]	< 0.0001
IL-6	5	248/248	130.43	97%	< 0.00001	Random-effects	Inverse Variance	−2.03[−3.30,-0.77]	0.002
TNF-α	6	303/303	38.24	87%	< 0.00001	Random-effects	Inverse Variance	−1.16[−1.66,-0.66]	< 0.00001

### Relationship of liraglutide with hypoglycemic related indicators and BMI

**Fig. 4:**
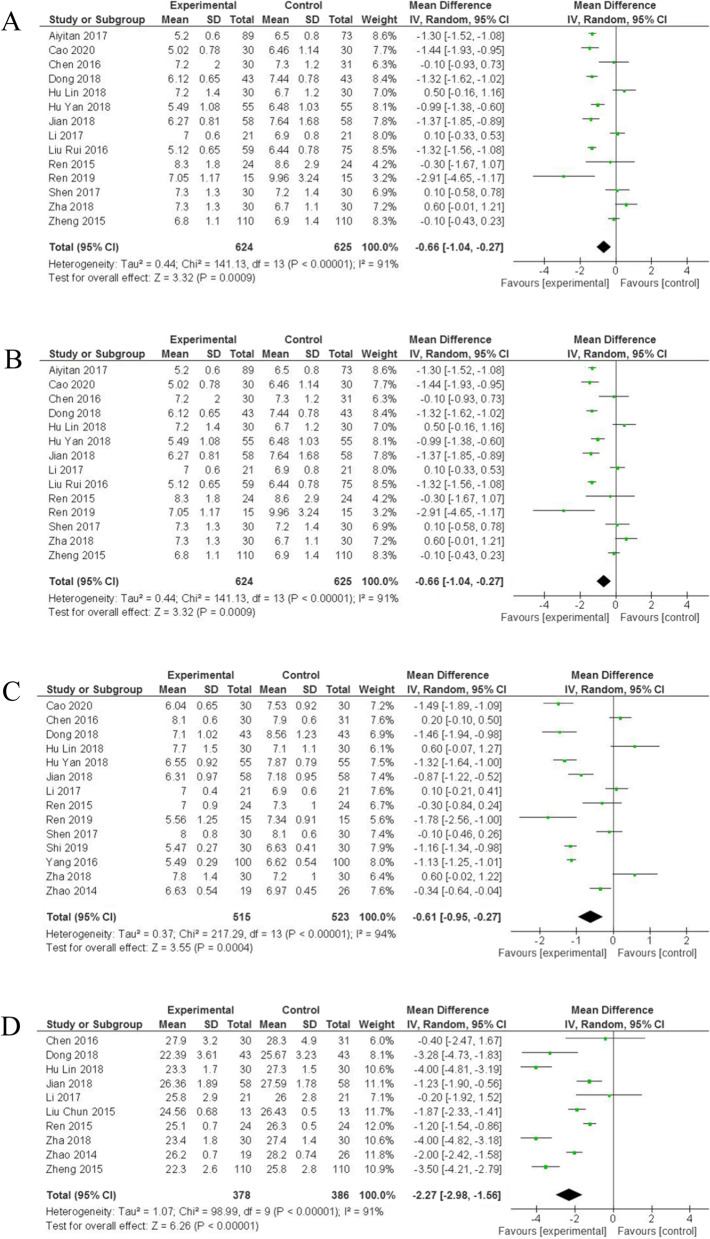
Forest plots for the effects of liraglutide on hypoglycemic-related indicators and BMI of patients with diabetic nephropathy. **A** FBG; **B** PBG; **C** HbA1c; **D** BMI. WMD, weight mean difference; CI, confidence interval; IV, inverse variance; df, degrees of freedom; green squares, an effect size of each study; the size of green squares, the weight of each study; Black diamonds, test for overall effect; horizontal lines, confidence intervals

For validating the influence of liraglutide on hypoglycemic-related indicators and BMI in patients with T2DN, the statistical differences of FBG, PBG, HbA1c, and BMI between the liraglutide group and the control group were estimated. As shown in the Fig. 4A and Table 2, the patients in the liraglutide group measured lower levels of FBG (WMD = -0.66, 95% CI: [− 1.04,-0.27], *p* = 0.0009) (Fig. 4A, Table 2), PBG (WMD = -1.51, 95% CI: [− 1.68,-1.34], *p* < 0.00001) (Fig. 4B, Table 2), HbA1c (WMD = -0.61, 95% CI: [− 0.95,-0.27], *p* = 0.0004) (Fig. 4C, Table 2) and BMI (WMD = -2.27, 95% CI:[− 2.98,-1.56], *p* < 0.00001) (Fig. 4D, Table 2) than patients in the control group, suggesting the excellent performance in controlling blood sugar and weight loss of liraglutide.

### Relationship of liraglutide with anti-inflammatory indicators

**Fig. 5:**
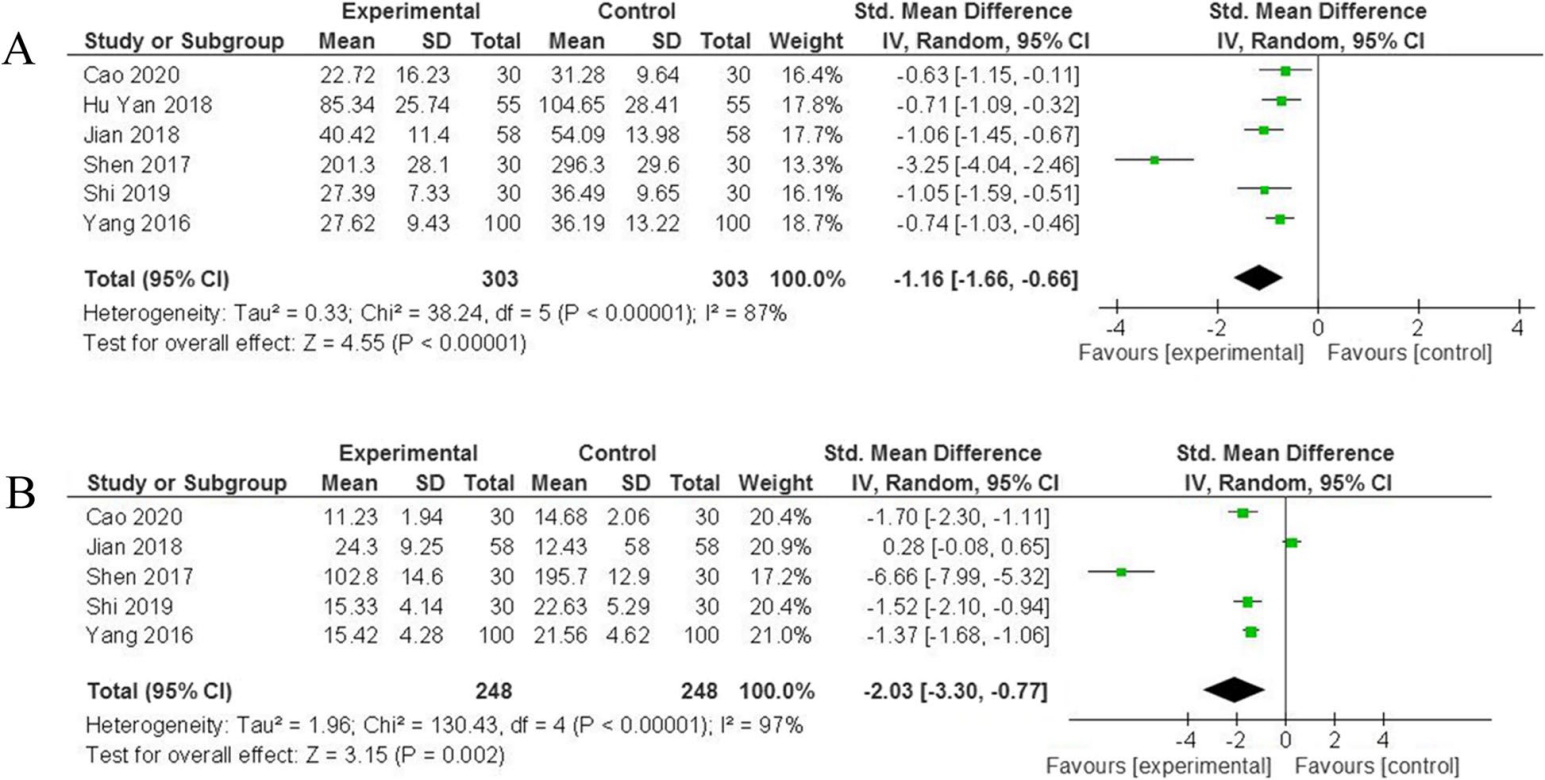
Forest plots for the effects of liraglutide on anti-inflammatory indicators of patients with diabetic nephropathy. **A** TNF-α; **B** IL-6. SMD, standard mean difference; CI, confidence interval; IV, inverse variance; df, degrees of freedom; green squares, the effect size of each study; the size of green squares, the weight of each study; Black diamonds, test for overall effect; horizontal lines, confidence intervals

To further confirm whether the liraglutide could reduce inflammatory reaction and thus prevent renal fibrosis, we compared the levels of IL-6 and TNF-α between the liraglutide group and the control group after drug intervention. Notably, liraglutide was demonstrated to delay the process of renal fibrosis by anti-inflammatory in our analysis. Obviously, the liraglutide group had lower level of TNF-α (SMD = -1.16, 95% CI: [− 1.66,-0.66], *p* < 0.00001) (Fig. 5A, Table 2) and IL-6 (SMD = -2.03, 95% CI: [− 3.30,-0.77], *p* = 0.002) (Fig. 5B, Table 2) than control group.

### Sensitivity analysis and evaluation of publication bias

**Fig. 6:**
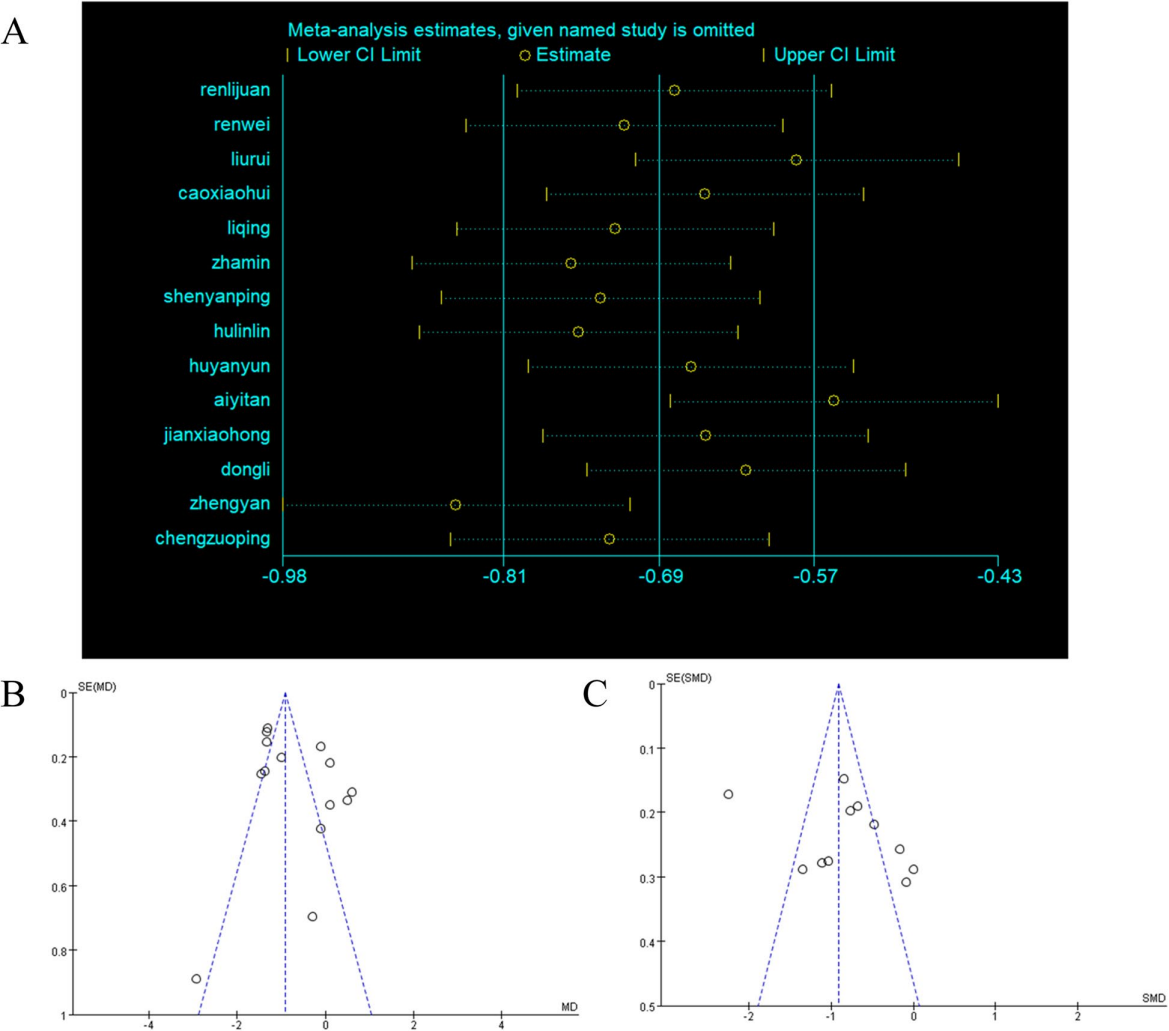
Sensitivity analysis and funnel plots for assessment of publication bias. **A** Sensitivity analysis based on FBG; **B** funnel plots based on FBG; **C** funnel plots based on Scr. MD, mean difference; SE(MD), Standard Error (mean difference); SE(SMD), Standard Error (standard mean difference)

To verify the sources of heterogeneity, sensitivity analysis by removing each study gradually was performed using Stata 13 software. The result showed that when removing the studies of Zhengyan [25] and Aiyitan [27], obvious changes of pooled WMD were found (Fig. 6A). Therefore, we considered the heterogeneity coming from these two studies. Moreover, funnel plots drawn with Revman software were used to show publication bias. Asymmetry was detected in Fig. 6B-C, suggesting that there may be publication bias, and the results that are not statistically significant may not be published.

## Discussion

Diabetic Nephropathy(DN) is one of the main complications of Diabetes Mellitus. DN is caused by a number of causes, including metabolic and hemodynamic disorders [34]. Some studies have shown that GLP-1RA can reduce proteinuria and improve renal function. The mechanism may be that GLP-1RA induce NHE3 (Na+ /H+ exchanger 3-) phosphorylated and activated, which can result in the reabsorption of filtered Na + increase in the proximal tubule [35, 36], which may improve renal hemodynamics in diabetes-associated glomerular hyperfiltration through overlapping and separate mechanisms, then helps to reduce albuminuria. Liraglutide also alleviated the accumulation of glomerular extracellular matrix (ECM) and renal injury in DN by improving the signaling of Wnt/β-catenin. The Wnt/β-catenin signaling pathway is involved in mesangial cell production of ECM (MCs). Treatment with liraglutide significantly reduced high glucose (HG)-stimulated production of fibronectin (FN), collagen IV (Col IV), and alpha-smooth muscle actin (alpha-SMA) in cultured human mesangial cells (HMCs) and significantly attenuated the liraglutide effects with XAV-939, a selective Wnt/β-catenin signaling inhibitor [37]. In addition, Our results have shown that liraglutide can reduce urinary protein indicators of UACR and renal function indicators including Scr and Cysc, which also confirm these points. But GLP-1RA has no clinically important effect on SUN and eGFR, which may be due to the insufficient number of RCT included.

About blood glucose and BMI, liraglutide decreased BMI and blood glucose levels in the current meta-analysis [38]. The hypoglycemic mechanism of liraglutide relies on it can increase insulin secretion and alpha beta-cell action to inhibit glucagon release, which leads to decreased plasma glucose in diabetes patients, and the role of central nervous receptors to increase satiety delayed gastric emptying [39]. Our results also show that liraglutide can decrease the level of blood glucose, BMI.

In the development of DN, NF-kB plays a central role in the inflammatory pathway [40]. Regulated nuclear factor kappa-b NF-kB activation and subsequent inflammatory response in mesangial cells are involved in the hyperglycemia-induced downregulation of GLP-1R [41]. In this meta-analysis, the results also showed that, in the liraglutide group, the down-regulated TNF-a and IL-6 levels were better than in the control group. Although the number of studies we included was small, liraglutide was shown to have an anti-inflammatory effect on the kidney in conjunction with previous studies.

The following limitations exist in this study. No blind method was used in all the studies. As a result, the quality of the included literature declined relatively, and there exists implementation bias. The inconsistency in baseline data, treatment base measures, and experimental protocols of Zhengyan’s and Aiyitan’s study led to heterogeneity according to our sensitivity analysis.

## Conclusion

In patients with DN, liraglutide appears to be beneficial in lowering urine protein, strengthening renal function, improving blood sugar levels, and having anti-inflammatory effects. To further investigate the effects of GLP-1RA liraglutide on ESRD in the future, and provide evidence-based medical information to prove clinical safety and rational drug usage, RCTs from more centers and large sample randomized double-blind controlled trials are needed due to certain limitations. Thus, liraglutide therapy of patients with type 2 diabetes has beneficial effects on kidney outcomes. Such findings support the advantages of using liraglutide for clinical use.

## Data Availability

There is no additional data than the contained within the present manuscript.

## Abbreviations

T2DN: Type2 diabetic nephropathy
CNKI: Chinese National Knowledge Infrastructure
CBM: China Biology Medicine Database
RCTs: Randomized controlled trials
BMI: Body mass index
Lira: Liraglutide
ESRD: End-stage renal disease
GLP-1RA: Glucagon-like peptide 1 receptor agonists
eGFR: Glomerular filtration rate
UACR: Urine albumin creatinine ratio
BUN: Blood Urea Nitrogen
Scr: Serum creatinine
CysC: Serum cystatin C
SMD: Standardized Mean Difference
WMD: Weighted Mean Difference
ECM: Extracellular matrix
HMCs: Human mesangial cells
FN: Fibronectin
Col IV: Collagen IV
NF-kB: Nuclear factor kappa-b
